# Cystic Fibrosis and New Trends by Ophthalmological Evaluation: A Pilot Study

**DOI:** 10.1155/2014/580373

**Published:** 2014-07-15

**Authors:** Marcella Nebbioso, Serena Quattrucci, Emanuela Leggieri, Leopoldo Spadea, Enzo Maria Vingolo

**Affiliations:** ^1^Department of Sense Organs, Ocular Electrophysiology Center, Policlinico Umberto I, Viale del Policlinico 155, 00161 Rome, Italy; ^2^Sapienza University of Rome, Piazzale A. Moro 5, 00185 Rome, Italy; ^3^Department of Pediatrics and Pediatric Neurology, Cystic Fibrosis Center, Policlinico Umberto I, Viale del Policlinico 155, 00161 Rome, Italy; ^4^Department of Ophthalmology, Polo Pontino, A. Fiorini Hospital, Via Firenze, 04019 Terracina, Italy

## Abstract

*Background.* Cystic fibrosis (CF) is characterized by hypoxia that affects several organic tissues. Retinal ganglion cells may suffer from the hypoxic status, and this may lead to alterations of retinal nerve fiber. *Methods.* Twenty-two eyes in CF patients were analyzed. A complete ocular evaluation and visual field exams of the 30 central degrees were performed using the frequency doubling technology (FDT). Forced expiratory volume in one second (FEV1%), forced vital capacity (FVC%), oxyhaemoglobin saturation (SpO_2_%), and hematocrit (Ht%) have been calculated. FDT analyzed parameters were mean deviation (MD) and pattern standard deviation (PSD). Pearson's correlation was chosen as statistical analysis. *Results.* Data showed statistically significant relationship between MD and Ht% (*r* value −0.18; *P* = 0.04), MD and FEV1% (*r* value −0.68; *P* = 0.001), and MD and FVC% (*r* value −0.45; *P* = 0.005). Moreover, there were correlations between PSD and Ht% (*r* value 0.29; *P* = 0.03), PSD and SpO_2_% (*r* value −0.31; *P* = 0.01), PSD and FEV1% (*r* value 0.71; *P* = 0.0005), and PSD and FVC% (*r* value 0.63; *P* = 0.003). *Conclusions.* The oxygen supply alterations might determine hypoxia of the ganglion cells causing a decrease of receptive optic nerve fiber activity. This method could be also useful to evaluate indirectly pulmonary activity of the CF disease.

## 1. Introduction

Cystic fibrosis (CF), also known as mucoviscidosis, is an autosomal recessive genetic disorder that affects most critically the lungs and also the pancreas, liver, and intestine. It is defined from many researchers as the most common and severe genetic disease in Caucasians [1–3]. CF is a multisystem disorder with a high morbidity and mortality from pulmonary and gastrointestinal tract diseases. In 1938, nearly 70% of babies died before the first year of life. Latest figures from the US Cystic Fibrosis Foundation show a 50% survival to 27.6 years. Fortunately, advances in care and therapies have led to an important increase in survival for these patients to nearly 40 years in 2012 from early childhood years in the 1940s [1–3].

This disease is characterized by abnormal transport of chloride/sodium and water across the epithelial surfaces in the gastrointestinal and respiratory tracts, the reproductive system, and the sweat glands, leading to thick, viscous secretions [3–6]. Acute or chronic hypoxemia due to changes of oxygen tension in healthy, older people or by chronic lung pathology can cause a polyneuropathy and likewise visual functionality alterations as seen in normal subjects or in patients affected from chronic obstructive pulmonary diseases (COPD) [7–12].

Rabe et al. had defined COPD as “a preventable and treatable disease with some significant extrapulmonary effects that may contribute to the severity in individual patients.”

The airflow limitation is usually progressive and associated with an abnormal inflammatory response of the lung to noxious particles or gases [13]. Consequently, optic nerve and retinal cells, in particular ganglion cells, may suffer from chronic hypoxic status, and this may damage the retinal nerve fibers. Successively, the hypoxic status may lead to alterations in the responses of the visual fields of CF patients.

To the best of our knowledge, the relation between COPD of subjects affected from CF and unconventional computerized perimetry has not been studied. The aim of our research has been to evaluate the functionality of the optic nerve and retinal ganglion cells by ocular examination in subjects affected by CF.

## 2. Methods 

The study protocol was approved by the Ethics Committees, and in accordance with Helsinki Declaration written consent was obtained for each subject or their parents.

Eleven patients (6 male and 5 female) were recruited within a group of individuals, stable at the time of the study and in good respiratory conditions, from CF Centre in Rome, Policlinico Umberto I, Sapienza University, Italy (Table 1). The diagnoses of the patients with COPD were established according to the GOLD Guidelines [13]. They were identified and enrolled by means of several criteria. Patients with asthma, interstitial lung diseases, tuberculosis, and bronchiectasis were excluded for possible fits of coughing that might have interfered with ocular fixation during the test. Moreover, subjects affected by medical problems, like neurological disorders, multiple sclerosis, epilepsy, systemic chronic diseases, diabetes, hypertension, ocular trauma, ocular diseases, optic neuritis, retinal pathologies, cataract, glaucoma, and high refractive errors, were excluded from the study for possible cross related interference with the test.

The criteria of inclusion were as follows:

- patients with diagnosis of CF confirmed by abnormal chloride concentrations (≥70 mmo L^−1^) in pilocarpine iontophoresis sweat test and the identification of mutation in the CFTR gene (cystic fibrosis transmembrane conductance regulator);
- FEV1%, 35–90% predicted;
- males or females aged from 14 to 56 years.

Data collected included demographics, medications, pulmonary function tests, and arterial blood gases. Airflow limitation was measured by spirometry that is the most widely available and reproducible test of lung function. In particular, the group was subjected to the following tests: forced expiratory volume in one second (FEV1%), forced vital capacity (FVC%), oxyhemoglobin saturation (SpO_2_%), hematocrit Ht%, and visual field examination of the 30 central degrees.

Then all the individuals were also analyzed in the Electrophysiological Ocular Centre of the Policlinico Umberto I. Twenty-two eyes in CF patients were subjected to complete ocular evaluation. All eyes presented visual acuity of 20/20 as detected using Snellen charts.

Visual field was performed using the Humphrey frequency doubling technology (FDT) Matrix perimetry (Welch Allyn, Skaneateles, NY; Zeiss Humphrey, San Leonardo, CA) after familiarization with the procedures to avoid learning effect.

During FDT recordings, all patients were seated comfortably in a semidarkened room. They were asked to keep their eye on a central fixation point when they were simultaneously exposed to the stimuli coming from a monitor for about 6 minutes with the right eye and, successively, for another 6 minutes with the left eye. Simultaneously, the patients were invited to press a button when they saw, with the eye open, the bars on the monitor. Global indices were evaluated like mean deviation (MD), pattern standard deviation (PSD), glaucoma hemifield test (GHT), central threshold of sensitivity, reliability, and testing time.

**Figure 1:**
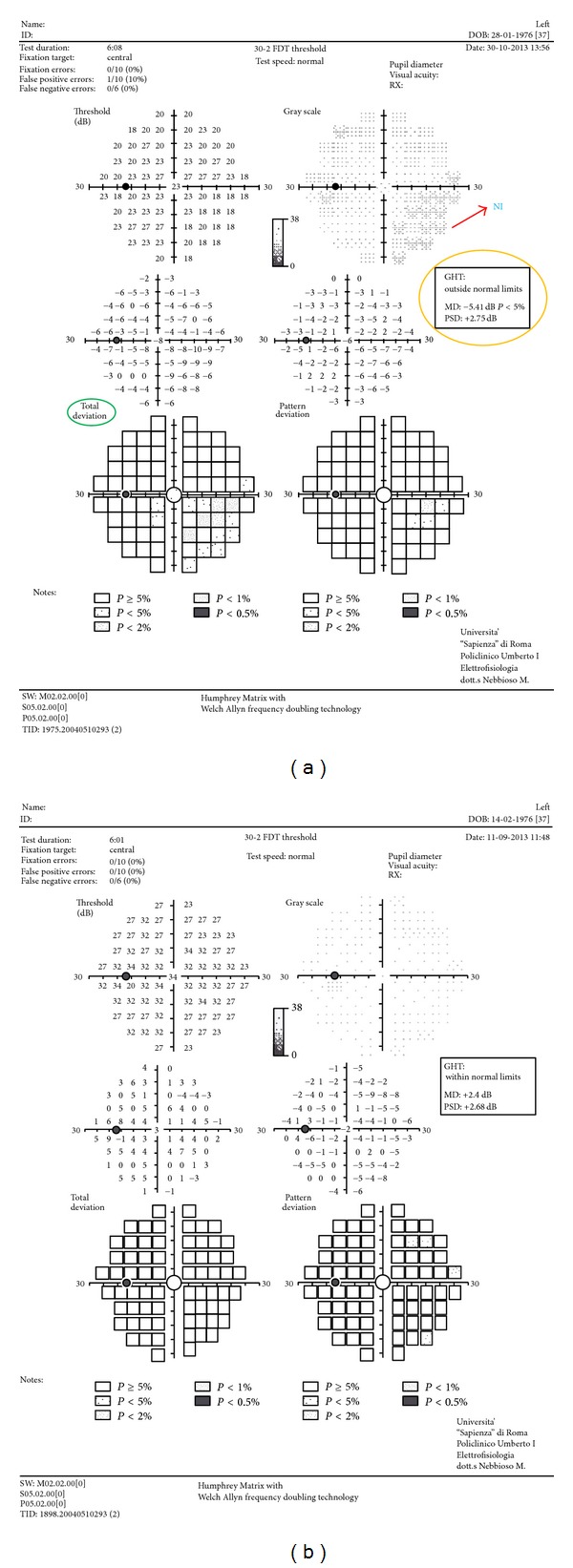
The output data from the frequency doubling technology (FDT) perimetry includes number, location, and severity of the defects in the 69 target zones. Readings appear as a numerical table and as probability maps with five gray tones. (a) Abnormal glaucoma hemifield test (GHT) and mean deviation (MD) at FDT perimetry with decreased average responses of a patient* versus* control subject from an age-normalized database (see total deviation). In particular, decreased average responses in nasal-inferior quadrant (NI); GHT: outside normal limits; and MD: −5.41 dB (*P* < 5%). (b) FDT of control.

The Matrix FDT perimeter uses a modified binary search scale Bayesian threshold estimation strategy known as ZEST, in which the contrast of the frequency doubling stimulus is increased if the target is not seen and decreased if the target is seen. A severe defect is reported when a target, presented for the fourth time, at maximum contrast, is not seen. The age of the subject is entered so that the instrument can choose expected values of contrast from an age-normalized database [14, 15]. The output data from the FDT perimetry includes number, location, and severity of the defects in the 69 target zones (Figure 1).

Humphrey* FDT Matrix perimeter* is a contrast threshold psychophysical test with a more complex target than the standard threshold automated perimetry. Its target consists of a low spatial frequency, 0.50 cycles per degree (cpd), in combination with a high temporal frequency stimulus, 18 Hz, and a mean luminance of 50 candelas/meter squared (cd/m^2^). Each target is 5°  × 5° square, except for the central target which is a 5° diameter circle. A video display unit presents the patient with a stimulus that is a monochrome sinusoidal grating of vertical black and white bars. These bars undergo rapid counterphase flickering (rate of 18 times per second). A total of 69 stimulus locations are shown, 17 in each quadrant and 1 in the macula for the 30-2 threshold program. Each target preferentially stimulates the magnocellular (M-cells) pathway [14, 15]. Readings appear as a numerical table and as probability maps with five gray tones.

### 2.1. Statistical Methods

Sample size was calculated by means of MedCalc software using the sampling function with correlation coefficient as input data. The highest Pearson's correlation coefficient (0.71), as obtained from preliminary data among optometric and respiratory parameters, along with error parameters type I (*α*) = 0.10 and type II (*β*) = 0.20, was employed. Using the highest correlation coefficient ensured the lowest number of subjects to be enrolled for this pilot study. Data obtained were used for subsequent statistical analysis. All results were expressed as mean ± SD when appropriate and were compared. Calculation of Pearson's correlation was chosen as statistical analysis. Parameters were performed to assess relationship between pulmonary function tests, arterial blood gases, and retinal activity. Statistical significance was set at *P* < 0.05. For the purpose of our study, according to other presented papers on the use of FDT, it was chosen to use both eyes in statistical analysis. Usually, FDT parameters represents locally determined ocular responses to contrast bar motion. In fact, there is no influence in the answers of the patients between the two eyes.

## 3. Results

Mean age of the 11 CF patients was 30 ± 13 SD years old (range of 14 to 56 years). Demographic characteristics, data of the pulmonary, and ocular functionality of cases were summarized in Table 1. The best corrected visual acuity of CF patients was 20/20. The intraocular pressure (IOP) was measured by means of Goldmann applanation tonometer (the reference ranges of the normal were 10–20 mmHg). Horizontal cup/disc ratio (optic disc cupping area/optic disc area) was measured by OCT scans. IOP and cup/disc were within the reference ranges of normality (Table 1).

The correlation of Pearson between all this data revealed an *r* value of −0.18 between MD and Ht%; an *r* value of −0.08 between MD and SpO_2_%; an *r* value of 0.29 between PSD and Ht%; an *r* value of −0.31 between PSD and SpO_2_% (Table 2).

Moreover the correlation of Pearson showed an *r* value of −0.68 between MD and FEV1%; an *r* value of −0.45 between MD and FVC%; an *r* value of 0.71 between PSD and FEV1%; an *r* value of 0.63 between PSD and FVC%. There were significant correlations between the mean values of the pulmonary and ocular functionality in CF patients (*P* < 0.05) (Table 2). No relationship was found between MD and SpO_2_ values. Whereas, the best significant statistical correlations were identified between parameters of the spirometry and visual field: FEV1, FVC, MD, and PSD.

## 4. Discussion

The objective of the present study was to determine the relationship between ocular activity and pulmonary functionality in CF patients. We investigated whether moderate COPD in our patients had any effect, in particular, on optic nerve and ganglion retinal cells by means of unconventional central visual field. It is unclear whether a relationship between retinal activity and maximal oxygen uptake exists in CF and, if so, whether the relationship reflects a direct effect or is mediated by the effects of confounding variables, such as pulmonary function. During repetitive episodes of hypoxemia and hypercapnia, the optic nerve may have a progressive decrease in functionality of the ganglion cells and successively of the retinal nerve fiber layer [16, 17]. Using FDT perimetry a significant correlation was found between pulmonary function tests, arterial blood gases, and the parameters of the visual field with preservation of the central visual acuity.

Several studies have shown that COPD is a major risk factor reducing tissue oxygen delivery and this effect may be responsible for the visual field abnormalities [13].

It is well known that FDT, unconventional perimetry, can identify defects earlier than standard static perimetry in glaucoma patients where there is some kind of underling ischemic deficit [14, 15]. Moreover, PSD reflects localized sensitivity loss and MD is associated with diffuse reductions in retinal sensitivity of the visual field. It is thought that FDT perimetry reflects the visual processing, mainly, via the magnocellular pathway and is a more demanding procedure than static perimetry [14, 15].

So flickering perimetry increases the metabolic demand and, consequently, the blood flow of the retina, but during retinal hypoxia the FDT responses would be reduced in CF patients. Moreover, the inner retinal is supplied with a blood flow with autoregulation. Hence, the loss of autoregulation in retinal blood vessels and the resulting chronic effects on the visual field could be caused from the pulmonary disease in CF patients.

We speculate that long term chronic hypoxia and vascular dysregulation even if not so deep could be the cause of damage of ganglion cells activity by disturbing inner retinal layers perfusion. This damage leads to optic nerve fibers dysfunction and could be cause of changes similar to the glaucomatous disease in the magnocellular system, undetectable to conventional computerized visual field. According to several authors a reduction in the ventilator drive, caused by hypoxia and hypercapnia, conduces to a decrease in pO_2_ and an increase in pCO_2_ [18–22]. Moreover, hypoxemia results in increased levels of the vasoconstrictor endothelin production. The endothelial cells also produce nitric oxide that is a vasodilator. A loss of ganglion cells activity could be also secondary to the imbalance between nitric oxide and endothelin-1 production [18–20].

However Kergoat et al. (2006) have demonstrated that ganglion cell function is reduced during mild hypoxia and suggested that the metabolic changes induced by hypoxia were not fully compensated by vascular regulation of the inner retina [23].

We detected that the initial defects appear to have similar characteristics to those of patients with glaucoma in which a kind of ischemia of the optic nerve is responsible for damage to visual field. This leads us to speculate on the fact that CF disease results, also initially, in a malfunction of magnocellular system as in early glaucoma or diabetic retinopathy damage.

Our findings have implications concerning the sensitivity of flicker perimetry in the early detection of ischemic/hypoxic retinal disorders and suggest that the development of new perimetric test protocols may increase sensitivity of visual perimetry to ischemic/hypoxic retinal disorders [24].

To conclude, we have hypothesized that some elements of the exams carried out could be used as parameters to value the current state of chronic hypoxia and help formulate new models of ascertaining of our patients. We think that FDT perimetry is able to highlight the defects of the visual field, in conditions of hypoxia also mild, even if further evaluation is necessary to define the effects of COPD on unconventional perimetry. In summary, we will continue the research on CF elderly patients to verify our hypothesis of the study considering ophthalmological alterations in different CF groups with mild/moderate/severe pulmonary function tests.

## Figures and Tables

**Table 1:** The baseline characteristics of 11 CF (cystic fibrosis) patients, 6 male and 5 female, are presented as mean ± SD. Glaucoma hemifield test (GHT) in FDT (frequency doubling technology) perimetry was borderline in 5/22, altered in 2/22, and normal in 15/22 eyes.

Parameter	Mean	SD (±)
Age (years)	29.91	13.01
IOP (mmHg)	14.1	2.5
VA (BCVA)	20/20	0.0
RE (sphere)	−1.05	2.31
Cup/disc (mm)	0.26	0.07
Ht (%)	43.88	4.59
SpO_2 _(%)	97.64	0.012
FEV1 (%)	58	0.15
FVC (%)	74.73	0.14
MD (dB)	1.36	1.81
PSD (dB)	2.84	0.53
CT (dB)	31.32	4.39
TT (minutes)	6.14	0.17

SD: standard deviation. IOP: intraocular pressure. VA: visual acuity. BCVA: best corrected visual acuity for far distance. RE: refractive error. Cup/disc: horizontal optic disc cupping area/optic disc area at slit lamp examination. Ht%: hematocrit. SpO_2_%: oxyhemoglobin saturation. FEV1%: forced expiratory volume in one second. FVC%: forced vital capacity. MD: mean deviation. PSD: pattern standard deviation. CT: central threshold. TT: test time.

**Table 2:** Pearson correlation coefficient (CC) results (parametric test). Statistical significance was set at *P* < 0.05.

Relationship	Pearson CC	*P* value
MD/Ht	−0.18	0.04
PSD/Ht	0.29	0.03
MD/SpO_2_	−0.08	NS
PSD/SpO_2_	−0.31	0. 02
MD/FEV1	−0.68	0.001
PSD/FEV1	0.71	0.001
MD/FVC	−0.45	0.005
PSD/FVC	0.63	0.003

MD: mean deviation. Ht%: hematocrit. PSD: pattern standard deviation. SpO_2_%: oxyhemoglobin saturation. FEV1%: forced expiratory volume in one second. FVC%: forced vital capacity. NS: not statistically significant.
